# The inflammatory and metabolic status of patients with sudden-onset sensorineural hearing loss

**DOI:** 10.3389/fneur.2024.1382096

**Published:** 2024-07-02

**Authors:** Jônatas Bussador do Amaral, Kelly Abdo Peron, Tracy Lima Tavares Soeiro, Marina Cançado Passarelli Scott, Flávia Tatiana Pedrolo Hortense, Michelly Damasceno da Silva, Carolina Nunes França, Luiz Henrique da Silva Nali, André Luis Lacerda Bachi, Norma de Oliveira Penido

**Affiliations:** ^1^ENT Research Lab, Department of Otorhinolaryngology—Head and Neck Surgery, Universidade Federal de São Paulo (UNIFESP), São Paulo, Brazil; ^2^Post-Graduation Program in Health Sciences, Santo Amaro University (UNISA), São Paulo, Brazil

**Keywords:** sudden sensorineural hearing loss, cytokines, inflammation, metabolism, adiponectin, IFNγ, TNF-α

## Abstract

**Introduction:**

Sudden sensorineural hearing loss (SSNHL) is a common emergency symptom in otolaryngology that requires immediate diagnosis and treatment. SSNHL has a multifactorial etiology, and its pathophysiologic mechanisms may be associated with inflammatory and metabolic changes that may affect the cochlear microenvironment or its nervous component, thus triggering the process or hindering hearing recovery. Therefore, the aim of this study was to assess metabolic and inflammatory changes to identify systemic parameters that could serve as prognostic factors for hearing recovery in patients with SSNHL.

**Materials and methods:**

Thirty patients with a sudden hearing loss of at least 30 dB in three contiguous frequencies were enrolled in this study. Patients were followed up for 4 months and peripheral blood samples were collected at 7 days (V1), 30 days (V2) and 120 days (V3). Interleukins (IL)-1F7, IL-2, IL-4, IL-5, IL-6, IL-10, interferon γ (IFN-γ), tumor necrosis factor α (TNF-α) and adiponectin were quantified in serum. In addition, lipid and glycemic profiles as well as concentration of creatinine, uric acid, fructosamine, peroxide, total proteins and albumin were analyzed. Patients underwent weekly ear-specific hearing tests with standard pure tone thresholds for frequencies of 250–8,000 Hz, speech recognition threshold and word recognition score.

**Results:**

Patients with SSNHL were divided into a group of patients who did not achieve hearing recovery (*n* = 14) and another group who achieved complete and significant recovery (*n* = 16). Most serologic parameters showed no significant changes or values indicating clinical changes. However, IFN-γ levels decreased by 36.3% between V1 and V2. The cytokine TNF-α showed a statistically significant decrease from V1 to V3 (from 22.91 to 10.34 pg./mL). Adiponectin showed a decrease from 553.7 ng/mL in V1 to 454.4 ng/mL in V3.

**Discussion:**

Our results show that serologic cytokine levels change in the acute phase of manifestation of SSNHL and establish a parallel between systemic changes and improvements in hearing, especially TNF-α, which showed differences in hearing recovery. The use of IFN-γ, TNF-α and adiponectin may elucidate the clinical improvement in these patients.

## 1 Introduction

Sudden Sensorineural Hearing Loss (SSNHL) is a common emergency symptom in otolaryngology that requires immediate diagnosis and treatment. It is clinically characterized by a rapid onset of sensorineural hearing loss of more than 30 dB in at least 3 contiguous audiometric frequencies within 72 h (1). SSNHL can usually manifest unilaterally, while in rare cases it can occur in both ears (simultaneously or sequentially) (2). The resulting deterioration in the quality of life of individuals affected by SSNHL may be exacerbated by other associated symptoms such as aural fullness, sound distortion, tinnitus, dizziness, and vertigo (3–5). Although a few cases are mild or resolve spontaneously, a minority of patients may develop permanent profound hearing loss associated with severe tinnitus and even vestibular symptoms (6). In some cases, SSNHL is diagnosed only by describing the symptoms of hearing loss; regardless of the cause, treatment methods are necessarily different (7). The prognosis for hearing recovery depends largely on the severity of hearing loss, age, time between onset of symptoms and treatment, comorbidities, and specific effects on cochlear structures (8–10).

SSNHL has a multifactorial etiology whose pathophysiologic mechanism remains unclear. The cause may be associated with cochlear membrane injury (11), microarteriosclerosis (12), microthrombosis (13), viral infections (14), autoimmunity (15), metabolic diseases (16) and other risk factors. The effect of these processes, which can even occur simultaneously, leads to inflammatory and metabolic changes that can affect the cochlear microenvironment or its nervous component. In this perspective, cytokines play an important role in the balance between innate and adaptive immunity (17). Their pro-inflammatory and anti-inflammatory effects may act on inflammatory and immunological processes both in the inner ear (18) and systemically (19) in the context of hearing loss. These compounds include interleukins (ILs), tumor necrosis factors (TNFs), interferons (IFNs), and adipocytokines, and can provide values prognosis and diagnosis in a variety of diseases and disorders, including neurological diseases, metabolic syndrome, cancer, and infectious diseases (20, 21). Inflammation is considered one of the significant causative factors for the occurrence of SSNHL, especially when analyzed in the context of vascular injury (22), endococlear responses (23), and atherogenesis (24).

Compared to the cochlear microenvironment, peripheral blood has easier access, and this approach is also used in studies related to hearing loss. The change in cytokine levels in the peripheral blood of patients with SSNHL indicates a possible systemic effect that may lead to lesions in the inner ear (19). An example of this is the decrease in IFN-γ and IL-12 levels and the increase in TNF-α levels and monocyte counts in patients with SSNHL compared to controls (25). Another interesting fact is that SSNHL patients with better prognosis had mononuclear cells that produced higher levels of IL-1β after activation with LPS (26).

Extending this analysis to other metabolic parameters makes the interpretation of SSNHL more thorough, as it also takes into account the additive effect of factors such as dyslipidemia (27), oxidative stress (28), hyperglycemia (29), and plasma proteins (30). For instance, high cholesterol levels have been associated with a higher incidence of SSNHL and poorer hearing recovery outcomes (31, 32). Changes in levels related to oxidative stress may also be altered in patients with SSNHL too. This ranges from an impaired thiol-disulfide balance to the possible formation of reactive oxygen species (ROS), which can affect microcirculation in the inner ear (33, 34). Glycemic changes can also have an impact on the prognosis of SNHL, as patients with poor glucose regulation have a poorer hearing outcome (35). In summary, a thorough understanding of the multiple functions of cytokines, lipids, glycemic factors and oxidative stress is essential to elucidate their complex role in physiological and pathological processes in SSNHL and may also provide new perspectives for the development of better treatment approaches.

The aim of this study was therefore to investigate the possible changes in oxidative stress, pro- and anti-inflammatory cytokines, protein, lipid and glucose profiles as well as over 7, 30, and 120 days in SSNHL patients undergoing treatment. In addition to comparing these results at these time points, the data will also be linked to hearing recovery observed.

## 2 Materials and methods

### 2.1 Participants, clinical data, and sample collection

Patients with a sudden hearing loss of at least 30 dB in three contiguous frequencies were included in this study and designated as having Sudden Sensorineural Hearing Loss (SSNHL). Exclusion criteria included congenital hearing loss, incomplete treatment, a diagnosis other than SSNHL (conductive hearing loss, acoustic trauma, vestibular schwannoma, Meniere’s disease) (36) and failure to follow-up. During a 4 month observation period, 30 patients with SSNHL were examined in the ENT emergency department and outpatient clinic of a tertiary hospital. During this period, audiometric examinations and blood samples were taken on three visit assessments: V1 took place after 7 days and allowed us to analyze the most acute phase of SSNHL involvement. V2 allowed us to follow the metabolic and inflammatory profile of the patients after 30 days of corticosteroid treatment, and finally V3, after 120 days, to obtain information on the recovery of hearing when no longer under corticosteroid treatment. Each patient underwent a thorough medical history, physical examination, audiometry and magnetic resonance imaging (MRI) of the inner ears (28). Only patients who were previously untreated were included in our case study. All participants provided written informed consent. The Ethics Committee for Research of the Escola Paulista de Medicina/Universidade Federal de São Paulo (EPM/UNIFESP) accepted the current study under protocol number 4.507.315.

### 2.2 Audiometric testes

Patients underwent weekly ear-specific auditory assessment with standard pure tone thresholds (PTTs) for frequencies of 250–8,000 Hz, speech recognition threshold (SRT) and word recognition score (WRS). Four sessions were carried out during the first 30 days, and then auditory assessments were moved to a monthly frequency until the completing 120 days. Tympanometry and acoustic reflex measurements were only performed at the first examination to exclude middle ear pathologies. The degree of HL was classified according to WHO 2020 using the four-frequency pure tone average (4fPTA) by taking the mean of the thresholds at 500, 1,000, 2,000, and 4,000 Hz (28). Hearing outcomes were analyzed in comparison between the first (7 days) and last (120 days) visit, with pure-tone and speech audiometer assessment based on the “Clinical Practice Guideline: Sudden Hearing Loss (Update)” (1). This particular guideline divides patients into three groups: Complete, partial or no recovery. To better allocate and for statistical analysis, the hearing recovery was subclassified in two groups: one comprising patients with complete and significant recovery and the other including patients with partial and no recovery. The first was defined as a hearing level difference of <10 dB between the affected and the unaffected ear and recovery of Word Recognition Scores (WRS) within 5 to 10% compared to the unaffected ear. Partial recovery was defined as 4fPTA < 50 dB or WRS > 50%, or an improvement of >10 dB in pure tone thresholds or an improvement in WRS ≥ 10%. Any hearing level improvement of <10 dB was classified as no recovery.

### 2.3 Corticosteroid treatment

Prednisolone 1 mg/kg/day (highest dose: 60 mg/day) was administered to all patients once daily for at least 14 days. Prednisolone was then reduced weekly until complete discontinuation within 15 days. In the following three weeks, the prednisolone dose was reduced until complete discontinuation. Patients who could not take prednisone and suffered from arterial hypertension or diabetes received equivalent doses of deflazacort (maximum dose 90 mg/day). Corticosteroid treatment was carried out throughout the first two samples (V1 and V2) (37). In the third collection (V3), all patients were no longer receiving corticosteroid treatment after 120 days.

### 2.4 Cytokines quantification

The serum was separated from the blood samples by centrifugation (800G, 8 min at 4°C) and stored at −80°C until analysis. The concentrations of interleukin (IL)-2, IL-4, IL-5, IL-6, IL-10, interferon γ (IFN-γ) and tumor necrosis factor α (TNF-α) were measured using ELISA kits (enzyme-linked immunosorbent assay) from Thermo Fisher Scientific (Vienna, Austria). IL-1F7 and adiponectin were determined using ELISA kits from RD Systems (Minneapolis, United States). The procedures were performed strictly according to the manufacturer’s protocols. Absorbance at 450 nm was measured with a Multiskan Sky Spectrophotometer microplate reader (Thermo Fisher Scientific—Vienna, Austria).

### 2.5 Metabolic parameters

Circulating serum levels of total cholesterol and fractions (LDL and HDL), triglycerides, albumin, total protein, creatinine, uric acid, fructosamine, glucose (LabTest diagnostica, Lagoa Santa, Brazil) and peroxide (Bioassay Systems-Hayward, United States) were determined with commercially available kits. The results were analysed using a Multiskan Sky Spectrophotometer microplate reader (Thermo Fisher Scientific—Vienna, Austria). LDL values were estimated according to the Friedewald formula (38).

### 2.6 Statistical analysis

Statistical analyses were calculated with SPSS v26.0 (IBM Corp). Graphs were created using GraphPad Prism v8.01 (GraphPad Software Inc.). The normality of the data was tested for normality using the Kolmogorov–Smirnov test. The comparison was analysed using the Wilcoxon test (for skewed continuous variables) or the Student *t*-test and the Anova one-way test (for normally distributed continuous variables). The correlation was calculated using Pearson’s rank correlation test. A *p* value < of 0.05 was considered statistically significant.

## 3 Results

### 3.1 Demographic data and clinical characteristics

The average age of the subjects was 50.26 years, the gender ratio was equal: 15 men and 15 women. The medical history revealed a variety of symptoms and diseases that had both local and systemic effects. Of particular note were systemic arterial hypertension (13 subjects), diabetes mellitus (7 subjects), chronic kidney disease (4 subjects) and hypothyroidism (3 subjects). Trigeminal neuralgia, herpes zoster, Raynaud’s disease and multiple myeloma each occurred once. In addition, the body mass index (BMI) was 27.37 kg/m^2^, a value indicative of overweight adults (39) (Table 1).

**Table 1:** Demographic data and clinical characteristics of the 30 subjects.

Variables	Value^*^
Female	15 (50%)
Age	50.26 ± 14.10 years (± SD)
BMI	27.37 ± 4.48 kg/m^2^ (± SD)
Tinnitus	30 (100%)
Vestibular symptoms	21 (70%)
Unilaterally affected ears	25 (83.33%)
Bilaterally affected ears	5 (16.67%)
Complete and significant hearing recovery**	16 (53.33%)
Partial and no hearing recovery**	14 (46.67%)
Systemic arterial hypertension	13 (43.33%)
Diabetes mellitus	7 (23.33%)
Chronic kidney disease	4 (13.33%)
Hypothyroidism	3 (10%)
Trigeminal neuralgia	1 (3.33%)
Herpes zoster	1 (3.33%)
Raynaud’s disease	1 (3.33%)
Multiple myeloma	1 (3.33%)

*Mean ± S.D. or *n* (%). **Classification based described by the American Academy of Otolaryngology—Head and Neck Surgery Foundation (AAO-HNSF) (1). SD, standard deviation.

**Figure 1:**
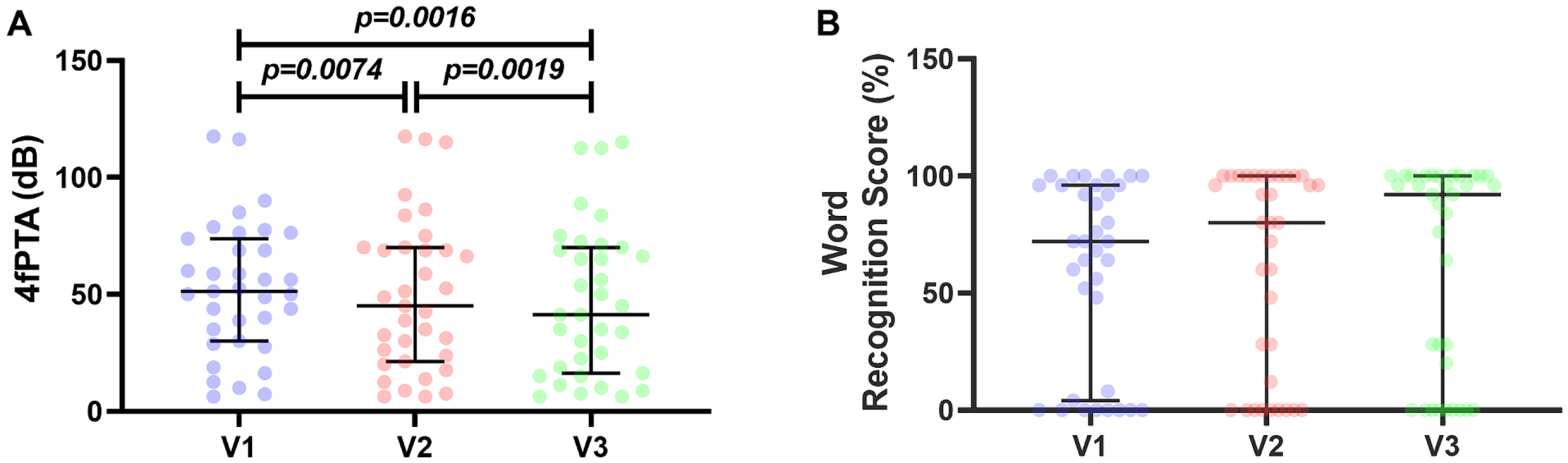
Audiometric evaluations of 30 patients followed up for 120 days. **(A)** Four-frequency pure tone average (4fPTA) in decibels (dB). **(B)** Word recognition score (WRS) in percentages (%). For statistical analysis, Wilcoxon test was used and *p*-values <0.05 were considered significant.

Four-frequency pure tone average (4fPTA) and the word recognition scores were carried out on all patients who took part in the study (Figure 1). Of the 30 study participants, 25 had unilateral SSNHL and 5 had bilateral SNHL (Table 1). For this reason, we analyzed all affected ears, totaling *n* = 35. The 4fPTA assessment showed that patients’ hearing deficits decreased over time between visits, with median values of 51.25 dB in V1, 45.00 dB in V2, and 41.25 dB in V3. On closer inspection, 6 subjects showed an increase in hearing threshold, with the worst case being a 67.5 dB increase in tone threshold. In four subjects, the hearing threshold remained the same, and in the remaining 25 subjects, the hearing threshold decreased in a range from 1.25 dB (which would be considered clinically unchanged) to patients who showed a decrease of 51.25 dB (63.75 to 12.5 dB) (Figure 1A).

Figure 1B shows the word recognition scores for the 3 visits analyzed. The median values of this test were: 72% in V1, 80% in V2 and 92% in V3. Regarding the other symptoms associated with hearing loss, all subjects in the study had tinnitus and 70% of them also had vestibular symptoms (Table 1).

### 3.2 Metabolic evaluation

**Figure 2:**
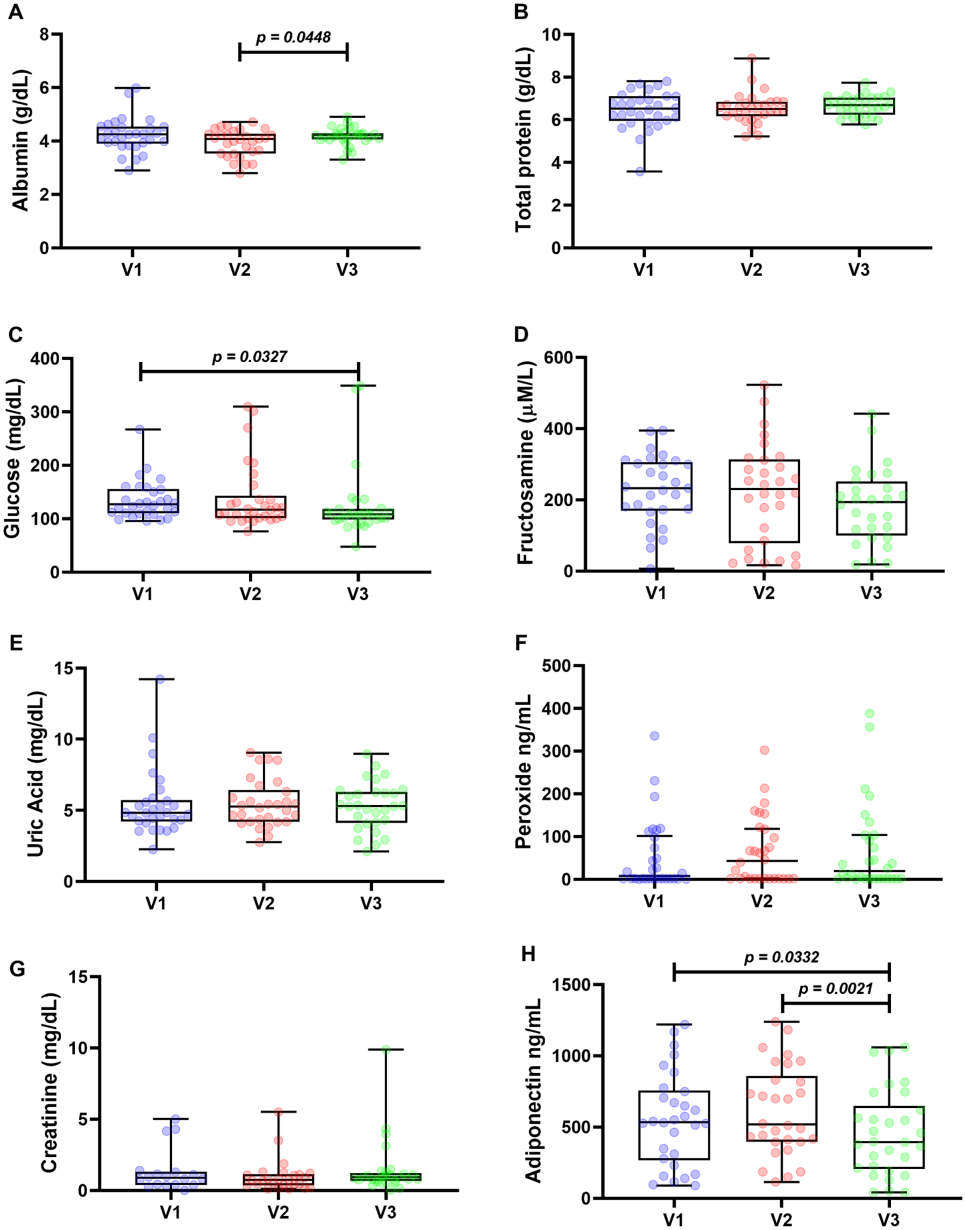
Metabolic assessments of 30 patients followed up for 120 days. Protein profile: albumin **(A)** and total proteins **(B)**. Glycemic profile: glucose **(C)** and fructosamine **(D)**. Uric acid **(E)**, peroxide **(F)**, creatinine **(G)**, and adiponectin **(H)** Wilcoxon test was used for statistical analysis in **(C)**, **(E–G)**, Anova one way **(A,B,H)**, and paired *t* test in **(D)**. Values of *p* < 0.05 were considered significant.

Mean albumin levels were similar, showing mean values of 4.232 g/dL in V1, 3.930 g/dL in V2, and 4.174 g/dL in V3. The only statistical difference was the increase between V2 and V3. There were no statistically significant differences in total protein, and the mean values were very close throughout the period. The mean values were 6.456 g/dL in V1, 6.535 g/dL in V2, and 6.671 g/dL in V3 (Figure 2B).

Glycemic parameters were determined by quantification of glucose and fructosamine (Figures 2C,D). Median glucose levels were 126.7 mg/dL in V1, 117.2 mg/dL in V2, and 108.4 mg/dL in V3, with only the decrease between V2 and V3 being statistically significant. Fructosamine results showed the following mean values: 227.4 μM/L in V1, 220.3 μM/L in V2, and 183.1 μM/L in V3, with no significant differences between visits.

Concentration of uric acid, peroxide, and creatinine showed no statistically significant differences (Figures 2E–G). The medians found for uric acid and creatinine were closest between time points, with uric acid values of 4.83 mg/dL in V1, 5.27 mg/dL in V2, and 5.30 mg/dL in V3, and creatinine of 1.359 mg/dL in V1, 0.991 mg/dL in V2, and 1.444 mg/dL in V3. Quantification of peroxide in serum showed results ranging from 0.400 to 388.1 ng/mL. Because of this wide range of values, the peroxide medians found were 7.90 ng/mL in V1, 43.05 ng/mL in V2, and 19.20 ng/mL in V3. Figure 2H shows the mean adiponectin values: 553.7 ng/mL in V1, 612.5 ng/mL in V2, and 454.4 ng/mL in V3. The decrease at V3 is statistically significant compared with the other visits.

**Figure 3:**
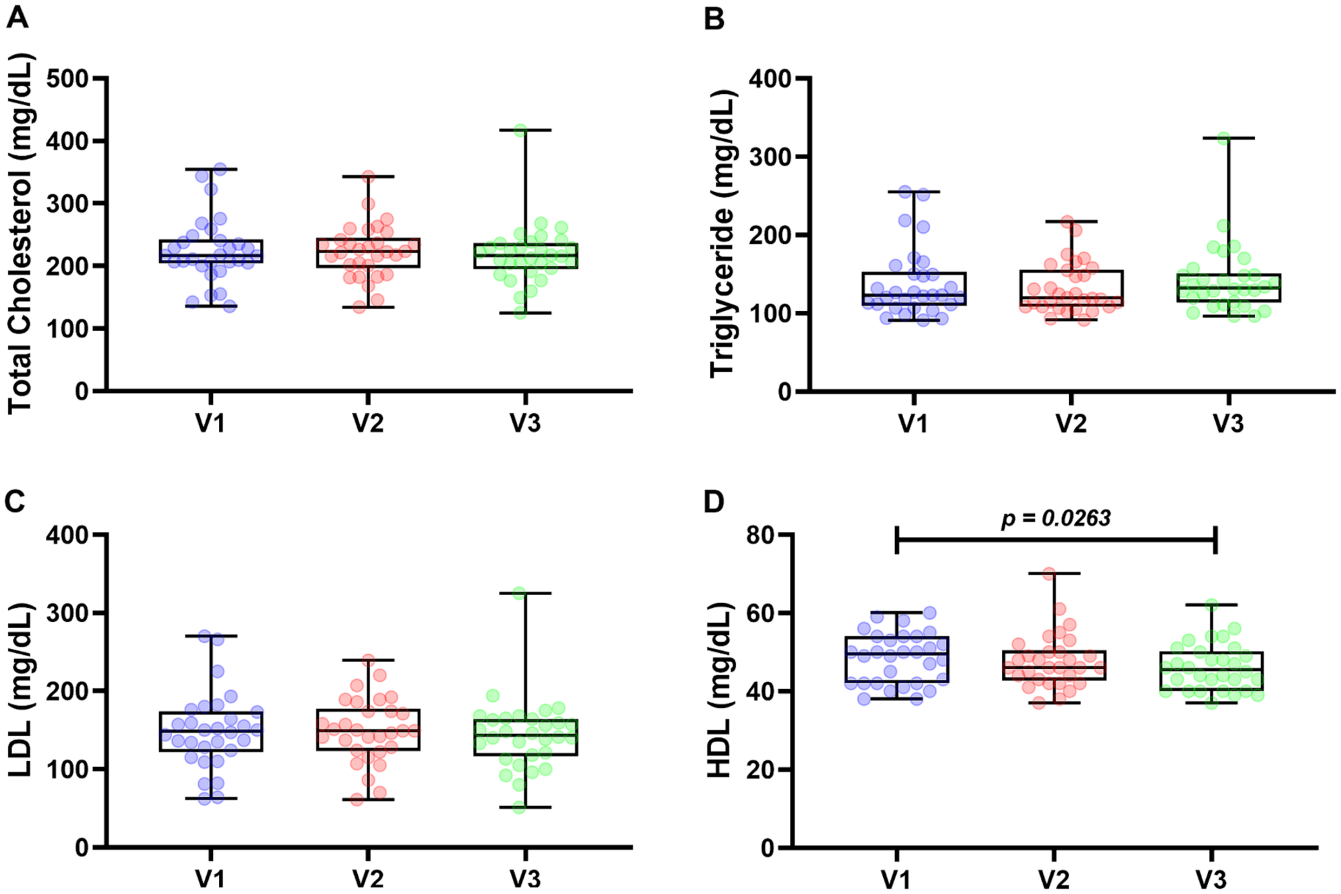
Lipid profile of 30 patients followed up for 120 days. Total cholesterol **(A)**, triglycerides **(B)**, LDL **(C)**, and HDL **(D)**. For statistical analysis, Wilcoxon test was used in **(A,B)**, and paired *t*-test was used in **(C,D)**. Values of *p* < 0.05 were considered significant.

Regarding the lipid profile analysis, total cholesterol, triglycerides, LDL, and HDL were quantified. HDL was the only analyte that showed statistically significant differences at each visit (Figure 3). Median total cholesterol levels were 216.7 mg/dL in V1, 223.1 mg/dL in V2, and 216.4 mg/mL in V3. The medians of triglycerides were 122.9 mg/mL in V1, 119.4 mg/mL in V2, and 132.3 mg/mL in V3. The LDL averages were 148.7, 149.3, and 144 mg/mL in V1, V2, and V3, respectively. Finally, mean HDL levels were 48.40 mg/mL in V1, 47.73 mg/mL in V2, and 46.07 mg/mL in V3. The only statistically significant difference was the reduction in values in V3 compared to V1.

### 3.3 Inflammatory cytokines

**Figure 4:**
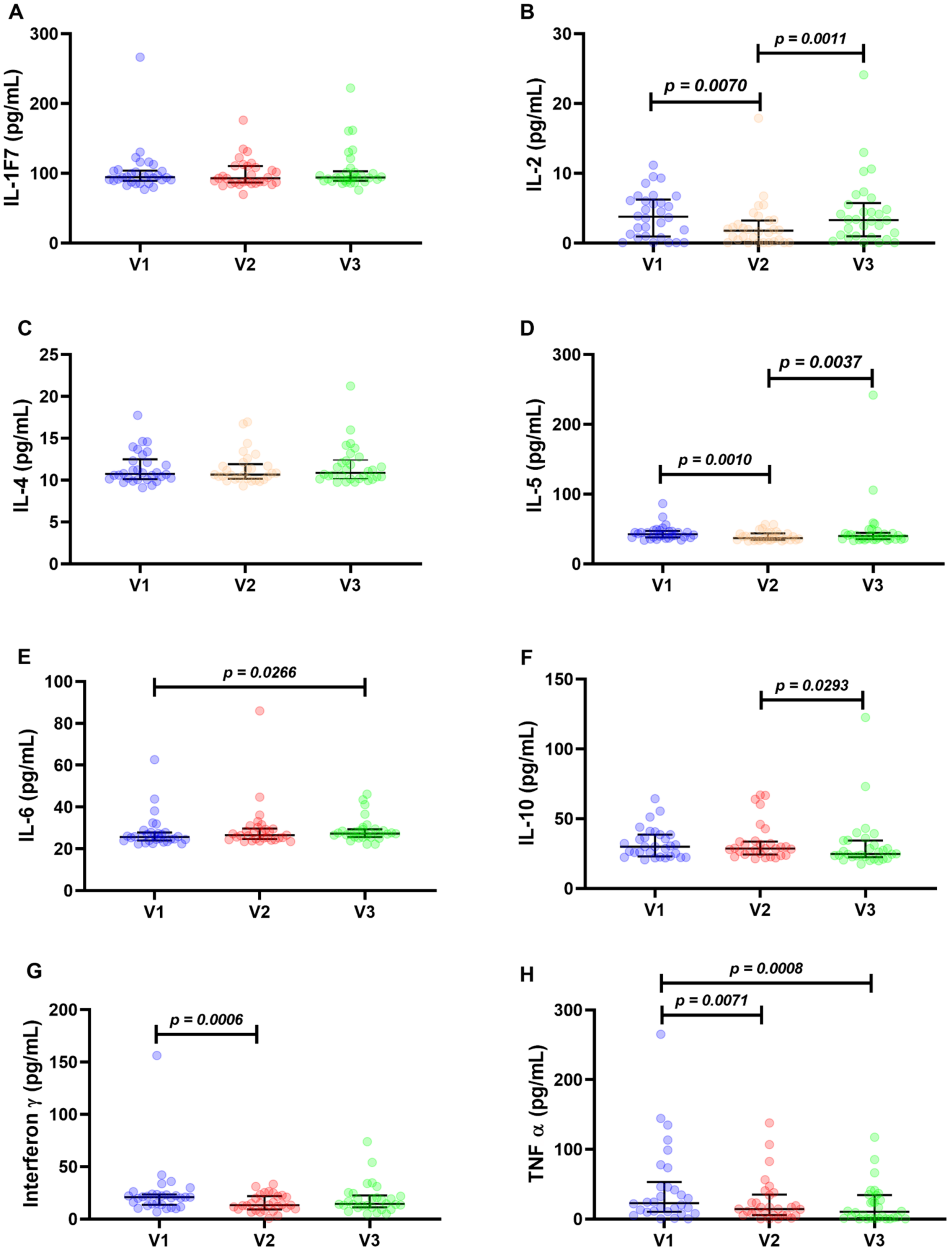
Inflammatory cytokines of 30 patients followed up for 120 days. Interleukin 1F7 **(A)**, interleukin 2 **(B)**, interleukin 4 **(C)**, interleukin 5 **(D)**, interleukin 6 **(E)**, interleukin 10 **(F)**, interferon γ **(G)**, and TNF-α **(H)**. The Wilcoxon test was used for statistical analysis, and *p* values <0.05 were considered significant.

Interleukins IL-1F7 (Figure 4A) and IL-4 (Figure 4C) showed no statistically significant differences. Their medians were: IL-1F7: 94.33 pg./mL in V1, 92.75 pg./mL in V2, and 93.96 pg./mL in V3. For IL-4 quantification: 10.73 pg./mL in V1, 10.74 pg./mL in V2, and 10.87 pg./mL in V3.

IL-2 dosages had the following medians: 3.75 pg./mL in V1, 1.75 pg./mL in V2, and 3.30 in V3. It is noteworthy that the levels of this interleukin decreased at visit 2, a statistically significant difference (Figure 4B). This decrease in V2 was also observed for IL-5, with medians of 42.25, 36.92, and 39.81 pg./mL in V1, V2, and V3, respectively (Figure 4D).

Interleukin 6 had the following medians: 26.66 pg./mL in V1, 26.43 pg./mL in V2, and 27.29 pg./mL in V3. Although the values were very close, there was a statistical difference at points V1 and V3 (Figure 4E).

Interleukin 10 showed a statistically significant decrease between V2 and V3, with median values of 29.93 pg./mL in V1, 28.52 pg./mL in V2, and 24.65 pg./mL in V3 (Figure 4F).

The Interferon γ decreased between V1 and V2, with median values of 20.94, 13.33, and 14.36 pg./mL in V1, V2, and V3, respectively (Figure 4G).

The cytokine TNF-α showed a statistically significant decrease from V1 to V2 (with a median decrease from 22.91 to 14.36 pg./mL) and from V1 to V3 (from 22.91 to 10.34 pg./mL) (Figure 4H).

### 3.4 Pearson’s correlation coefficient (*ρ*)

Supplementary material shows the different correlations between the results presented in Table 1 and Figures 1–4.

The 4fPTA showed a negative correlation coefficient with the Word Recognition Score. This correlation was very strong as it was present in the 3 analyzed periods with the following values: *r* = −0.947 in V1, *r* = −0.939 in V2, and *r* = −0.935 in V3. Both 4fPTA and the Word Recognition Score (WRS) also showed a relationship with peroxide in V2 (*r* = −0.446 for 4fPTA and 0.410 for WRS).

The correlation between total cholesterol X LDL was maintained across the 3 visits, with *ρ* values of *r* = 0.947 in V1, *r* = 0.942 in V2, and *r* = 0.945 in V3. The correlation between total cholesterol X HDL was observed only in V1 and V3, with values of *r* = 0.665 and 0.450, respectively. Total cholesterol also showed a correlation with adiponectin in V2 (*r* = 0.435) and had a negative correlation coefficient with some interleukins in V2, which values were: *r* = −0.487 for IL-2, *r* = −0.456 for IL-4, and *r* = −0.366 for IL-6.

The analysis of cholesterol fractions also allowed to establish correlations. The correlation HDL X LDL occurred only in V1 and showed *ρ* values of *r* = 0.480. HDL showed correlations with the interleukins IL-2 (*r* = 0.439 in V1) and IL-4, the latter with negative values (*r* = −0.404 in V3). This cholesterol fraction also correlated with Word Recognition Score (*r* = 0.408 in V2) and peroxide (*r* = 0.395 in V3). HDL levels correlated negatively with 4fPTA (*r* = −0.389 in V2). In contrast, the LDL fraction correlated negatively with IL-6 in V1 (*r* = −0.395) and V3 (*r* = −0.413). In the latter period, LDL also correlated with IL-2 (*r* = −0.434) and IL-4 (*r* = −0.438). Adiponectin and total proteins correlated with LDL only in V2, with values of *r* = 0.470 and 0.378, respectively.

The correlation with triglycerides that remained the same at all 3 time points was that produced by peroxide, with the coefficient values found being: *r* = 0.451 (V1), 0.504 (V2), and 0.362 (V3). The correlation with interleukins was IL-5 in V1 (*r* = 0.442) and IL-6 in V1 (*r* = 0.668) and V2 (*r* = 0.446). The correlation coefficient between triglycerides and age was *r* = 0.397 in V1, and in V2 the results showed a negative correlation coefficient in relation to fructosamine (−0.412) and 4fPTA (*r* = −0.400).

With the exception of IL-1F7, all other inflammatory cytokines showed correlations with each other, with interleukins 4 and 5 being more pronounced. It is noteworthy that these correlations are present eight times in V1, decrease to two correlations among cytokines in V2, and increase again to five correlations in V3. The only correlation that remained the same at all three time points was that of IL-4 X IL-2, which showed a decrease in the intensity of correlations between V1 and V2 (*r* = 0.472 and *r* = 0.435, respectively), with this coefficient increasing in V3 (*r* = 0.682). The correlation of TNF-α with IL-4 and IL-5 was present in V1 and V3, with values for IL-4 X TNF-α being very close at these two time points, *r* = 0.599 in V1 and *r* = 0.605 in V3. The correlation IL-5 X TNF-α gave values of 0.568 in V1 and 0.518 in V3. The interferon-γ correlation is also present in IL-4 and IL-5. However, these correlations occur only in V1. The value of IL-4 X interferon-γ was *r* = 0.642. The correlation between IL-5 and interferon-γ was *r* = 0.586. Interleukins 4 and 5 yielded a correlation coefficient (*r* = 0.374) only in V3. Interleukin 4 also correlated with IL-10 in V1 (*r* = 0.374). Another cytokine that correlated with IL-10 was TNF-α (*r* = 0.410 in V1). TNF-α also correlated with the following cytokines: Interferon-γ (*r* = 0.492 in V1); and IL-2 in V3, where the correlation coefficient was negative (*r* = −0.363).

Albumin and total protein also correlated with other parameters analyzed. Albumin, for example, had a negative correlation coefficient with IL-4 in V1 (*r* = −0.382) and in V2 (*r* = −0.371). The correlations also extended to fructosamine in V1 (*r* = 0.476) and established a correlation with a negative coefficient with TNF-α (*r* = −0.421 in V1), IL-2 (*r* = −0.373 in V1), and finally with uric acid in V3 (*r* = 0.555). Total proteins also showed a negative personal correlation coefficient, correlating with IL-6 (*r* = −0.361), IL-4 (*r* = −0.506), and fructosamine (*r* = −0.386) in V2.

Adiponectin also correlated with other parameters: IL-10 in V2 (*r* = 0.390) and, with negative correlation coefficients, with uric acid (*r* = −0.497 in V1), peroxide (*r* = −0.494 in V3), and body mass index (*r* = −0.405 in V3).

Uric acid also correlated with other parameters such as IL-1F7 (*r* = 0.404 in V3) and showed negative correlation coefficients with TNF-α (*r* = −0.364 in V2) and IL-5, this cytokine showing a correlation in V1 and V2 (*r* = −0.337 and *r* = −0.478 respectively).

Glycemic responses were also correlated with other parameters. Fructosamide was correlated with age (*r* = 0.565 in V1) and INF-γ in V2. Glucose also showed a correlation with peroxide (*r* = 0.457 in V2).

Finally, a correlation was found between BMI and TNF-α, which was correlated with negative coefficients in V1 (*r* = −0.377) and V2 (*r* = −0.412).

### 3.5 Outcome analysis on hearing recovery

**Figure 5:**
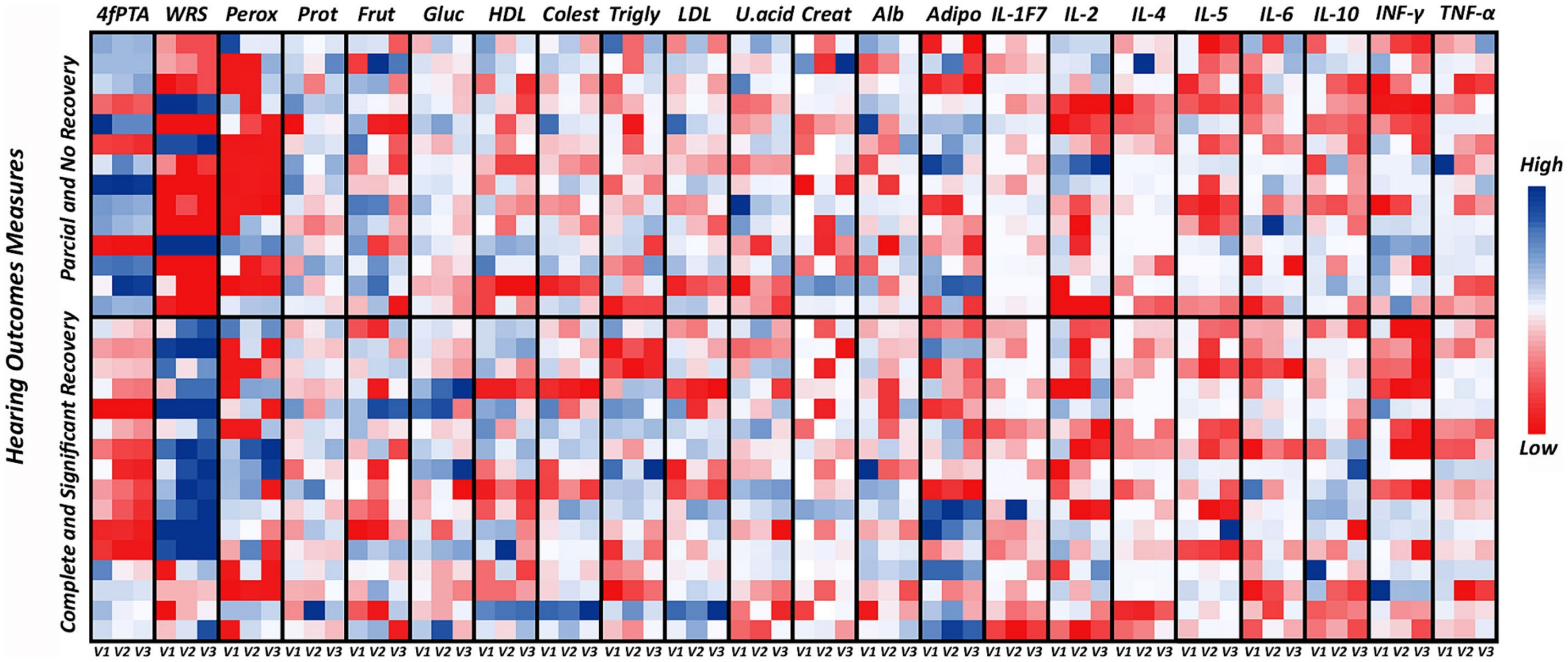
Heat map of metabolic and inflammatory parameters in patients with sudden sensorineural hearing loss. The values for each patient are shown in the small squares, with the color intensity representing the highest values in blue, the average values in white and the lowest values in red for each analyte tested after 7 days (V1), 30 days (V2) and 120 days (V3). The graph is divided according to hearing recovery, with 14 patients having partial and no recovery and 16 having complete and significant hearing recovery. V1 = 7 days, V2 = 30 days and V3 = 120 days. 4fPTA, four-frequency pure tone average; WRS, word recognition score; Perox, peroxide; Frut, frutosamine; Gluc, glucose; Colest, cholesterol; Trigly, triglycerides; U. acid, uric acid; Creat, creatinine; Alb, albumin; Adipo, adiponectin.

Due to the sudden onset of hearing loss, all patients had hearing characteristics that made them suitable for the study. Over time and as a result of the treatment, we observed statistically significant changes in hearing, metabolic and inflammatory parameters at the three proposed visits. This analysis was complemented by the results in terms of hearing recovery at the end of the study (120 days). After analyzing the results in Table and Figure 1, the patients were divided into a group of patients with partial and no hearing recovery (*n* = 14) and another group who achieved complete and significant hearing recovery (*n* = 16). Figure 5 shows all metabolic and inflammatory parameters when analyzed the hearing recovery. Significant variations between 4fPTA, WRS and peroxide can be observed when comparing the two groups. Tables 2, 3 show the variations between visits, which showed statistically significant differences. Table 2 shows the results of patients who had complete and significant hearing recovery. As expected, 4fPTA and WRS scores improved over time, with the median 4fPTA going from 46.87 db in V1 to 31.87 db in V3 and the WRS score increasing from 82 to 96% (V1 and V3, respectively). Compared to the results in patients who had not recovered their hearing, both 4fPTA and WRS scores showed no statistically significant differences between visits, with median 4fPTA of 71.25, 69.68 and 70.62 db and WRS of 28, 6 and 10% over V1, V2 and V3, respectively. One observation that stands out is the arrangement of the peroxide results in Figure 5. When all data were analyzed together (Figure 2F), no statistically significant differences were found between visits. However, when the patients were divided into 2 groups, the lowest values (in red in Figure 5) were concentrated in the patients for whom hearing did not recover. The medians for this group were 1.65 in V1, 1.7 in V2 and 20.25 ng/mL in V3 and were not statistically different. However, for the patients who showed complete and significant hearing recovery, the medians were 15.8, 66.55 and 19.2 ng/mL (V1, V2 and V3, respectively). In addition to the higher median values, the increase at 30 days was statistically significant in patients who showed some hearing improvement (Table 2).

**Table 2:** Parameters with statistically significant differences between patients with hearing recovery (complete and significant).

4fPTA	Db	*p*-value*
V1 (7d)	46.87 (31.56; 58.125)	0.001 (V2-V1)
V2 (30d)	32.5 (15.31; 47.81)	0.001 (V3-V1)
V3 (120d)	31.87 (12.18; 44.06)	0.007 (V3-V2)
WRS	%	*p*-value*
V1 (7d)	82 (64;95)	0.012 (V2-V1)
V2 (30d)	94 (80;100)	0.002 (V3-V1)
V3 (120d)	96 (89;100)	0.013 (V3-V2)
Peroxide	ng/mL	*p*-value*
V1 (7d)	15.8 (1.625; 108.22)	0.035 (V2-V1)
V2 (30d)	66.55 (27.22; 159.22)	0.836 (V3-V1)
V3 (120d)	19.2 (1.55; 139.2)	0.501 (V3-V2)
Adiponectin	ng/mL	*p*-value*
V1 (7d)	556.8 (286.14; 926.77)	0.570 (V2-V1)
V2 (30d)	569.4 (352.35; 926.77)	0.079 (V3-V1)
V3 (120d)	394.95 (240.39; 745.87)	0.026 (V3-V2)
IFN-γ	pg/mL	*p*-value*
V1 (7d)	18.71 (13.02; 28.30)	0.010 (V2-V1)
V2 (30d)	13.63 (9.98; 21.47)	0.121 (V3-V1)
V3 (120d)	14.26 (12.73; 23.30)	0.301 (V3-V2)
IL-2	pg/mL	*p*-value*
V1 (7d)	3.61 (1.05; 6.56)	0.031 (V2-V1)
V2 (30d)	1.57 (0.082; 2.975)	0.326 (V3-V1)
V3 (120d)	3.2 (0.8275; 6.04)	0.30 (V3-V2)
IL-5	pg/mL	*p*-value*
V1 (7d)	41.12 (38.36; 47.18)	0.015 (V2-V1)
V2 (30d)	38.83 (34.38; 44.25)	0.032 (V3-V1)
V3 (120d)	40.67 (35.21; 42.39)	0.127 (V3-V2)
TNF-α	pg/mL	*p*-value*
V1 (7d)	21.07 (11.31; 39.99)	0.026 (V2-V1)
V2 (30d)	12.4 (5.49; 36.9)	0.001 (V3-V1)
V3 (120d)	3.73 (0.41; 26.69)	0.016 (V3-V2)

*n* = 16. Data represent medians (interquartile interval 25; 75). *Wilcoxon test between visits. *p*-values < 0.05 were considered significant.

**Table 3:** Parameters with statistically significant differences between patients with partial and no recovery hearing.

Adiponectin	ng/mL	*p*-value*
V1 (7d)	529.05 (215.74; 716.17)	0.245 (V2-V1)
V2 (30d)	517.5 (415.12; 849)	0.300 (V3-V1)
V3 (120d)	418.35 (150.11; 569.85)	0.048 (V3-V2)
IFN-γ	pg/mL	*p*-value*
V1 (7d)	20.94 (17.48; 23.13)	0.048 (V2-V1)
V2 (30d)	12.5 (8.11; 22.72)	0.397 (V3-V1)
V3 (120d)	15.39 (10.47; 24.71)	0.331 (V3-V2)
IL-2	pg/mL	*p*-value*
V1 (7d)	4.17 (0.58; 6)	0.116 (V2-V1)
V2 (30d)	1.93 (0.36; 4.59)	0.650 (V3-V1)
V3 (120d)	3.75 (1.07; 5.95)	0.019 (V3-V2)
IL-5	pg/mL	*p*-value*
V1 (7d)	43.98 (36.47; 47.06)	0.048 (V2-V1)
V2 (30d)	36.24 (34.37; 43.89)	0.638 (V3-V1)
V3 (120d)	38 (35.69; 49.29)	0.016 (V3-V2)
Albumin	g/dL	*p*-value*
V1 (7d)	4.16 (3.72; 4.64)	0.272 (V2-V1)
V2 (30d)	4.04 (3.52; 4.27)	0.510 (V3-V1)
V3 (120d)	4.24 (4.16; 4.35)	0.041 (V3-V2)
Total Protein	g/dL	*p*-value*
V1 (7d)	6.86 (6.16;7.44)	0.177 (V2-V1)
V2 (30d)	6.51 (6.077; 6.75)	0.638 (V3-V1)
V3 (120d)	6.77 (6.50; 7.05)	0.048 (V3-V2)
IL-6	pg/mL	*p*-value*
V1 (7d)	26.46 (23.76; 28.88)	0.363 (V2-V1)
V2 (30d)	26.74 (24.39; 31.14)	0.035 (V3-V1)
V3 (120d)	28.23 (26.31; 37.70)	0.638 (V3-V2)
IL-10	pg/mL	*p*-value*
V1 (7d)	29.93 (22.32; 35.73)	0.778 (V2-V1)
V2 (30d)	28.62 (23.51; 34.45)	0.026 (V3-V1)
V3 (120d)	24.13 (22.5; 29.19)	0.019 (V3-V2)
HDL	mg/dL	*p*-value*
V1 (7d)	49 (42.75; 52.5)	0.156 (V2-V1)
V2 (30d)	45.5 (41.75; 49)	0.014 (V3-V1)
V3 (120d)	45.5 (40; 48)	0.592 (V3-V2)
Triglycerides	mg/dL	*p*-value*
V1 (7d)	124.84 (110.51; 136.51)	0.551 (V2-V1)
V2 (30d)	117.44 (107.88; 126.37)	0.414(V3-V1)
V3 (120d)	133.56 (117.40; 164.08)	0.035 (V3-V2)
Fructosamine	μmol/L	*p*-value*
V1 (7d)	275.32 (178.15; 324.42)	0.975 (V2-V1)
V2 (30d)	246.98 (197.89; 364.40)	0.026 (V3-V1)
V3 (120d)	122.48 (86.54; 208.52)	0.004 (V3-V2)
Glucose	mg/dL	*p*-value*
V1 (7d)	123,80 (112,25; 140,62)	0.198 (V2-V1)
V2 (30d)	116,70 (105,31; 128,99)	0.019 (V3-V1)
V3 (120d)	108,99 (100,46; 113,64)	0.019 (V3-V2)

*n* = 14. Data represent medians (interquartile interval 25; 75). *Wilcoxon test between visits. *p*-values < 0.05 were considered significant.

Adiponectin, interferon γ, IL-2, and IL-5 showed differences between visits in terms of hearing recovery regardless of situation. The decrease in adiponectin in V3 compared to V2 was maintained in all scenarios of this study. After 120 days, the values in patients with complete and significant hearing recovery fell from 569.4 ng/mL to 394.95 ng/mL (Table 2). In patients partian and no hearing recovery, the median values also fell in V3, with a concentration of 517.5 ng/mL in V2 and 418.35 ng/mL in V3 (Table 3). Interferon γ maintained its pattern of decline after the acute phase of SSNHL in all situations. The median levels found decreased from V1 to V2 as follows: 18.71 to 13.63 pg./mL in patients with complete and significant hearing recovery (Table 2) and 20.94 to 12.5 pg./mL in patients who did not recover their hearing (Table 3). IL-2 showed a significant reduction at visit 2 in all situations tested (Figure 4B and Tables 2, 3). In the group that showed complete and significant hearing recovery, the median levels at the 3 visits were 3.61, 1.57, and 3.2 pg./mL, respectively. In the patients who did not recover, the values were 4.17, 1.93, and 3.75 pg./mL. Statistical significance between visits varied, with only the increase from V2 to V3 being statistically significant in the group without hearing improvement. In the group with some hearing improvement, both the reduction in V2 and the subsequent increase in median levels in V3 were statistically significant (just as in the no separation group, Figure 4B). IL-5 showed too a very similar median values before and after separation according to hearing recovery. However, the statistical significance changed between visits, and in patients with complete and significant hearing recovery (Table 2), the statistically significant values were found in V2 and V3 compared to V1. The IL-5 medians in this context were 41.12 (V1), 38.83 (V2) and 40.67 pg./mL in (V3). The patients who did not regain their hearing had values of 43.98, 36.24, and 38 pg./mL (V1, V2 and V3, respectively), although there was no statistical difference when the values of V3 were compared with V1 (Table 3).

TNF-α showed a significant reduction over 120 days in the patients who showed complete and significant hearing recovery, from 21.07 pg./mL in V1 to 3.73 pg./mL in V3 (Table 2). This result is close to that found in the analysis of all 30 patients. However, in this case the statistical difference between V2 and V3 was not significant. When looking at the TNF-α levels in the patients who did not regain their hearing, no statistical difference was found between the visits. The median values in this group were: 28.42 in V1, 16.86 in V2, and 17.35 in V3.

The group that did not recover (Table 3) had statistically significant differences in some metabolic and inflammatory parameters that differed from those of the patients who recovered. However, when we looked at the differences between the visits, many values showed very little variation and were within normal parameters. Let us start with the blood protein levels (albumin and total protein). Only the values obtained at visit 3 showed statistical differences, and these values are practically the same as the other points. The median albumin values were 4.16, 4.04, and 4.24 g/dL and the total protein values were 6.86, 6.51, and 6.77 g/dL (V1, V2 and V3, respectively). The cytokines IL-6 and IL-10 as well as HDL also showed results that did not differ significantly from the values found in the 30 patients. Furthermore, in the cases where the values are statistically different, the results are very close to each other and have no clinical relevance. The median IL-6 levels were 26.46, 26.74, and 28.23 pg./mL (*p* = 0.035 for the difference between V1 and V3). IL-10, on the other hand, showed a decrease in V3 and the median values were 29.93, 28.62, and 24.13 pg./mL (*p* = 0.026 between V1 and V3 and *p* = 0.019 between V2 and V3). Finally, HDL levels were 49 in V1 and 45.5 in V2 and V3, with this decrease being statistically significant from V1 to V3. Another component of the lipid profile, triglycerides, showed an increase in the blood of patients in V3 (compared to V2), with medians of 124.84, 117.44, and 133.56 mg/dL (Table 3). Finally, for two glycemic parameters (fructosamine and glucose), values decreased between V2 and V3 (V1-V3 and V2-V3 with a statistical difference), with values of 275.32, 246.98, and 122.48 μmol/L for fructosamine and 123.80, 116.70, and 108.99 mg/dL for glucose (Table 3).

## 4 Discussion

It is well known that SSNHL is a condition with a number of causes, including autoimmune diseases, infections, trauma, as well as vascular, hematologic, and other factors (40). There is no recognized pathogenesis for SSNHL and this symptom is probably due to a multifactorial etiology. The results found are related to this, as they show that inflammatory and metabolic factors change during the development of SSNHL, demonstrating a dynamic interaction between the patient’s general condition, hearing recovery, treatment duration, and the effect of cytokines.

Identifying preventable or modifiable factors for hearing loss should be considered a top public health priority, given its potential impact on physical, mental and social well-being and quality of life in general. This would be of crucial importance in efforts to prevent or at least delay the onset of this disease (41). Obesity is an important factor as it can affect sensory systems and other organs, either directly or as a result of associated comorbidities, so an unhealthy metabolic status poses an additional risk (42). In this context, a first layer of analysis focused on factors related to fat metabolism. The mean body mass index of participants affected by SSNHL was described as overweight (27.37 ± 4.48 kg/m^2^). Studies show that high BMI levels may be associated with hearing loss (41), and obesity may be related to atherogenic processes that restrict blood flow in the cochlea, the release of proinflammatory cytokines by macrophage infiltrates, and hypoxia and oxidative stress which may negatively affect the innervation and hair cells in the cochlear microenvironment (42). In addition, adipose tissue plays an important endocrine function mediated by adipokines (43). Adiponectin is an adipokine that acts as a mediator of obesity-related metabolic and vascular diseases, and its imbalance can also affect hearing. The presence of the adiponectin receptor in the inner ear and the use of adiponectin-knockout mice have demonstrated its otoprotective role (44). Increased apoptosis of auditory sensory hair cells and endothelial cells has been associated with a decrease in adiponectin. Low adiponectin levels are also associated with reduced blood flow in the cochlea (45, 46). Our results show that after 120 days, the average adiponectin levels in all patients with SSNHL have decreased compared to visits 1 and 2. These visits (30 days) are the most acute period of manifestation of SSNHL. In our opinion, adiponectin could therefore act systemically and protect as an anti-inflammatory agent (47), as it showed a positive correlation with IL-10 at visit 2 (*r* = 0.390). The situation was similar with IL-10, which was also reduced in V3. The correlations with peroxide (*r* = −0.494) in V3 and with LDL (*r* = 0.470) in V2 and uric acid (*r* = −0.497) in V1 indicate a dynamic of protective relationships that changes during the course of SSNHL. It may act as a modulator of oxidative stress (48), as an LDL binder (49) or protection from uric acid-induced inflammation (50). No direct otoprotective effect can be derived from our results. However, the systemic effect of adiponectin could contribute to a clinical improvement and, consequently, better hearing recovery.

Analysis of the other metabolite parameters revealed an increase in albumin (V3) and a decrease in glucose and HDL (V3). Even when these differences are analyzed, the average values are very close to each other, which we do not consider to be a reduction in the relevant clinical value. Thus, we can assume that the average values of all metabolic parameters over the 120 days of the study showed no significant differences in their mean values. The interpretation of these results must be accompanied by a description of some pre-existing diseases in patients with SSNHL. Of the 30 patients analyzed, 13 had systemic arterial hypertension, 7 had diabetes mellitus and 4 had chronic kidney disease. So if we include BMI and age in this context, even if serologic analysis shows no significant abnormalities, the clinical profile suggests that metabolic imbalances may be present and acting on SSNHL. Previous studies by our group have shown that microangiopathies are more common in patients with SSNHL when diabetes mellitus, arterial hypertension and dyslipidemia are also present (51). Other factors such as chronic kidney disease (52), history of myocardial infarction and a higher risk of stroke in SSNHL (compared to controls) (53) suggest a possible vascular involvement in the its pathogenesis. Changes in the microstructure of the stria vascularis, hyperviscosity, alterations in endothelial function and the formation of atherosclerotic plaques are processes that play an important role in hearing loss (3). From our results, we can conclude that hyperlipidemia may not be acutely involved in the pathogenesis of SSNHL, as the lipid profile did not change during the study. The effects of the lipid profile leading to lesions in the inner ear take longer to manifest and are likely to be progressive and may act synergistically with inflammatory processes that may culminate in SSNHL.

Inflammation is a critical component in the pathogenesis of SSNHL (54, 55). Understanding this process at local and systemic levels is fundamental to a better understanding of symptoms, prognosis and hearing recovery. Therefore, systemic inflammatory markers may be useful for monitoring the development and progression of SSNHL over time (56, 57). In this context, this study investigated cytokines with pro-inflammatory and anti-inflammatory effects during the course of SSNHL over 120 days. In an initial analysis, 2 cytokines showed no changes across the 3 visits, namely IL-1F7 and IL-4. Interleukin 6 did show a statistically significant increase between visit 1 and visit 3, but the mean values found were very close to each other (26.66 pg./mL in V1 and 27.29 pg./mL in V3), which led us to believe that there was no change over time. Interleukin (IL) 1F7, also known as IL-37, has an important anti-inflammatory and immunosuppressive effect. Systemic alterations of IL-1F7 have been described in cancer (58), central nervous system disorders (59) and other autoimmune and inflammatory diseases (60). Although IL-1F7 has an effective effect on pro-inflammatory cytokines such as TNF-α, IL-6, and IL-1β (61), our results showed that IL-1F7 did not change at any of the time points analyzed and showed no correlation with other inflammatory cytokines. Only at visit 3 did it show a correlation with uric acid (*r* = 0.404), a result that was probably not related to SSNHL. In contrast to IL-1F7, the TH2 cytokines IL-4 and IL-6 have already been described as being involved in processes associated with SSNHL (26, 62). It is noteworthy that although our results showed no significant changes over the course of the visits, both cytokines showed a positive correlation at visit 2 (*r* = 0.547). The presence of these cytokines indicates pro-inflammatory processes, with IL-6 being associated with the response to infection and tissue damage (63) and IL-4 with the production of immunoglobulin E, regulation of cell proliferation and apoptosis (62). Our results suggest that IL-4 correlates not only with IL-6 but also with other pro- and anti-inflammatory cytokines across the 3 visits. The stimulation of IL-4 production in the context of SSNHL could therefore correlate with the general clinical condition of affected patients and interact with various inflammatory and metabolic aspects. For example, IL-4 shows a correlation with IFN-γ and TNF-α at visit 1 (*r* = 0.642 and 0.599, respectively), which would indicate a joint effect of proinflammatory cytokines in the acute phase of SSNHL. The results of the correlation with IL-2 at visit 1, 2 and 3 (*r* = 0.472, 0.435, and 0.682, respectively) suggest a common effect of different inflammatory showing a higher correlation in visit 3. However, the variability of interactions with other cytokines/metabolites also allows the interpretation that IL-4 is related to other processes not exclusively associated with SSNHL. This suggests that IL-4 may play a wide-ranging role in the maintenance of immune regulation and does not change between visits. Although the systemic presence of IL-4 has been described in patients with SSNHL (62), the function of this cytokine in the inner ear is still unknown. The effect of interleukin-6, on the other hand, has been described both in peripheral blood and in cochlear studies. For example, the relationship between IL-6 and the activation of the NF-kB signalling pathway has been described, as well as the relationship between the risk of vascular occlusion and the action of this cytokine (64), an increase in lesions in the human blood-labyrinth barrier model (65) or even an improvement in inflammatory parameters when this IL-6 cytokine is blocked in animal models (66). The quantification of this cytokine in peripheral blood still shows contradictory results, and although some studies have shown changes in this cytokine during the course of SSNHL (64), no direct correlation has yet been established between the levels found and disease progression (67). The possible correlation with IL-4 in V2 could indicate a synergistic effect within an immunoregulatory system. However, the fact that IL-6 shows no significant serologic changes over time could mean that its effect is limited to the inner ear in patients with SSNHL.

Interleukins 2 and 5 showed a decrease after 30 days with a subsequent increase in V3. These cytokines, which act through different pathways (IL-2 in TH1 and IL-5 in TH-2), are associated with hearing loss in a few articles. Interleukin 2 is secreted by activated T lymphocytes (both CD4+ and CD8+) and plays an important role in the proliferation of T and B lymphocytes by inducing effector T lymphocytes as well as generating Tregs that can prevent autoimmunity (68). In rats injected with IL-2 into the inner ear (round window), sensorineural hearing loss gradually developed within 5–7 days. Although it proved to be reversible, the inflammatory process impaired cochlear function (69). Interleukin-2 activates the endothelial cells of the modiolar spiral vein in the area of the cochlea so that they increasingly express ICAM-1 and take on the characteristics of high endothelial venules. These morphological changes allow the recruitment of leukocytes from the bloodstream, which in some situations trigger an inflammatory process that may be accompanied by the formation of fibrosis, which, if not absorbed, can form a fibro-osseous matrix that eventually leads to degeneration of the inner ear (70, 71). The association between IL-2 levels and progression of SSNHL has been described in patients treated with corticosteroids for 8 days (64), but it was not possible to discern a pattern between fluctuations in IL-2 levels during SSNHL treatment and disease progression. Our results are based on a longer analysis period (120 days), with corticosteroid treatment lasting 30 days. It is possible that this reduction in IL-2 levels in V2 is related to a systemic regulatory effect that may have been influenced by the corticosteroid treatment rather than an effect in the cochlea. IL-5 is produced by TH2 lymphocytes, mast cells and innate lymphoid cells. When activated by various environmental stimuli, these cells release this interleukin, which promotes eosinophil activation, maturation, survival and migration (72). The quantification of IL-5 in the patients in this study aimed to characterize a possible link between the worsening of inflammatory parameters (especially those related to allergens) and SSNHL. Interestingly, this cytokine maintained a significant positive correlation with IFN-γ and TNF-α in V1 (*r* = 0.586 and 0.568, respectively), which then disappeared in V2. The positive correlation is resumed later, after 120 days, with TNF-α (*r* = 0.586). These results suggest that systemically IL-5 acts synergistically with other inflammatory pathways that may influence the progression of SSNHL. Locally, no patient had middle ear manifestations of eosinophilic otitis, a condition strongly associated with the action of eosinophils and IL-5 (73).

Interleukin-10 plays a key role in the modulation of inflammation and the maintenance of cellular homeostasis. Its anti-inflammatory effect protects the body from an uncontrolled immune response. Its immunomodulatory role leads to important effects in diseases caused by a hyperinflammatory state, such as infectious diseases or cancer (74). It is increasingly recognized that inflammation in the cochlea contributes to the pathophysiology of sensorineural hearing loss. The local effect of IL-10 is evidenced by the presence of labeled cells in different regions of the inner ear following the induction of inflammatory processes by lipopolysaccharides in animal models (75). The quantification of IL-10 in the peripheral blood of patients affected by SSNHL has already been described in some studies, but this cytokine did not play a relevant role (25, 76). In our context, it is noteworthy that IL-10 levels decrease after 120 days, i.e., after the acute inflammatory process. Its correlation with adiponectin in V2 and especially with TNF-α in V1 (*r* = 0.410) suggests that the anti-inflammatory effect might be present throughout the treatment/time and has a systemic immunomodulatory effect during this period.

TNF-α is a cytokine with important key functions in various cellular processes, such as the maintenance of cellular homeostasis and the regulation of pro-inflammatory responses (77) and which plays an important role in the inner ear in the context of hearing loss (78). This cytokine may act in signaling pathways related to cell death (79), the process of differentiation of monocytes into mature dendritic cells (25), blood flow in the cochlea (80), and also in noise-induced hearing loss (81). Our results suggest that serum TNF-α levels decrease significantly between the acute phase (median 22.91 pg./mL) and V3 (median 10.34 pg./mL). In addition, the correlations with other cytokines (IL-2, 4, 5, 10 and IFN-γ) suggest that in the context of SSNHL, systemic TNF-α may be a parameter that should be analyzed during treatment. Some results support our findings, such as the fact that patients with immune-mediated sensorineural hearing loss had TNF-α levels above 18.8 pg./mL, with a greater than 97% positive predictive value (82). The reduction of TNF-α over time/treatment has also been described in patients with SSNHL between days 1 and 8, with a strong correlation between positive therapeutic outcomes and TNF-α reduction (64). In our analysis, we categorized the patients according to the outcome in terms of hearing recovery (Tables 2, 3). Interestingly, TNF-α levels decreased over time in patients who showed some improvement in hearing. However, in patients who did not recover their hearing, the levels did not differ significantly between visits. These results suggest a possible effect of TNF-α in relation to hearing recovery. The heterogeneity of the levels found for TNF-α in the peripheral circulation of patients with SSNHL (57, 76, 83) makes it necessary to further investigate the role of this cytokine, from the nature of its action (systemic and local effect) to its role in SSNHL (main effect or as part of an inflammatory chain).

IFN-γ is produced by NK cells and CD4 and CD8 T cells. One of the main functions of IFN-γ is the activation of macrophages to enhance phagocytosis, tumoricidal activity and intracellular clearance of pathogens, especially bacteria and fungi. Reactive oxygen and nitrogen intermediates and other inflammatory mediators are produced by macrophages in response to IFN-γ (84). Similar to the levels found for TNF-α, IFN-γ also showed a significant decrease between V1 and V2 in all patients with SSNHL. It was noteworthy that these two cytokines showed a positive correlation (*r* = 0.492) in the most acute phase of the process (V1). These results are consistent with those previously described in animal models evaluating inner ear injury, where IFN-γ locally increases the susceptibility of cochlear sensory cells to TNF-α cytotoxicity via JAK1/2-STAT1 signaling and caspase-1 activation (85). Its positive correlation with IL-4 and IL5 in the most acute phase of SSNHL demonstrates its importance for systemic inflammatory processes.

The complexity of the factors and the possibly different etiopathogenesis, which are still classified as idiopathic, may play a role in the progression of SSNHL. This means that the investigative approach is becoming broader, suggesting that early clinical phenotyping is necessary to select the appropriate laboratory tests to be performed in the etiologic investigation of this symptom. Our aim was to assist such selection by describing several inflammatory and metabolic parameters and relating them to hearing improvement over 120 days in patients with SSNHL.

Our results show that there is a considerable change in serologic cytokine levels in the acute phase of manifestation of SSNHL and a parallel can be established between systemic changes and improvements in hearing, especially when analyzed over time and as a result of outcomes related to hearing improvement. The use of IFN-γ, TNF-α and adiponectin may shed light on the clinical improvement in these patients, as these cytokines play a role in both the onset and 120 days of the study. The effect of TNF-α stands out because its modulation is not only part of the context of SSNHL implantation, but also of a possible differential effect in hearing recovery. So far, it has not been possible to identify a single biomarker that covers the multi-etiology of SSNHL symptoms. Further research into the role of inflammatory cytokines could be useful to obtain information on the relationship between systemic parameters and the inner ear and consequently to understand hearing recovery.

## Data availability statement

The raw data supporting the conclusions of this article will be made available by the authors, without undue reservation.

## Ethics statement

The studies involving humans were approved by the Ethics Committee for Research of the Escola Paulista de Medicina/Universidade Federal de São Paulo (EPM/UNIFESP) under protocol number 4.507.315. The studies were conducted in accordance with the local legislation and institutional requirements. The participants provided their written informed consent to participate in this study.

## Author contributions

JA: Conceptualization, Data curation, Formal analysis, Funding acquisition, Investigation, Methodology, Project administration, Writing – original draft. KP: Conceptualization, Investigation, Methodology, Writing – review & editing. TS: Conceptualization, Investigation, Methodology, Writing – review & editing. MaS: Conceptualization, Investigation, Methodology, Writing – review & editing. FH: Conceptualization, Investigation, Methodology, Writing – review & editing. MiS: Data curation, Formal analysis, Visualization, Writing – review & editing. CF: Data curation, Formal analysis, Visualization, Writing – review & editing. LN: Data curation, Formal analysis, Visualization, Writing – review & editing. AB: Conceptualization, Data curation, Formal analysis, Visualization, Writing – review & editing. NO: Conceptualization, Data curation, Formal analysis, Funding acquisition, Investigation, Methodology, Supervision, Visualization, Writing – original draft, Writing – review & editing.
