# Supplementary material for: The inflammatory and metabolic status of patients with sudden-onset sensorineural hearing loss
Source: Front Neurol. 2024 Jul 2;15:1382096. doi: 10.3389/fneur.2024.1382096 (PMC11250376; doi:10.3389/fneur.2024.1382096)
Supplement: Supplementary file 1 [file Table_1.DOCX]

Supplementary Material

| **Parameters** | **Visit 1** | | **Parameters** | **Visit 2** | | **Parameters** | **Visit 3** | |
| --- | --- | --- | --- | --- | --- | --- | --- | --- |
|  | ***r-value*** | ***p-value*** |  | ***r-value*** | ***p-value*** |  | ***r-value*** | ***p-value*** |
| **Albumin X Fructosamine** | *0.476* | *0.0091* | **Adiponectin X IL-10** | *0.390* | *0.0329* | **Adiponectin X BMI** | *-0.405* | *0.0293* |
| **Albumin X IL-2** | *-0.373* | *0.0425* | **Adiponectin X LDL** | *0.470* | *0.088* | **Adiponectin X Peroxide** | *-0.494* | *0.0056* |
| **Albumin X IL-4** | *-0.382* | *0,0372* | **Albumin X IL-4** | *-0.371* | *0.0433* | **Albumin X Uric Acid** | *0.555* | *0.0014* |
| **Albumin X TNF-α** | *-0,421* | *0.0206* | **Cholesterol X Adiponectin** | *0.435* | *0.0163* | **Cholesterol X IL-2** | *-0.487* | *0.0064* |
| **Cholesterol X HDL** | *0.665* | *<0.0001* | **Cholesterol X LDL** | *0.942* | *<0.0001* | **Cholesterol X IL-4** | *-0.456* | *0.0113* |
| **Cholesterol X LDL** | *0.947* | *<0.0001* | **Fructosamine X IFN-γ** | *0.371* | *0.0436* | **Cholesterol X IL-6** | *-0.366* | *0.0465* |
| **Fructosamine X Age** | *0.565* | *0.0014* | **Fructosamine X Triglycerides** | *-0.412* | *0.0238* | **Cholesterol X LDL** | *0.945* | *<0.0001* |
| **HDL X LDL** | *0.480* | *0.0073* | **Glucose X Peroxide** | *0.457* | *0.0111* | **HDL X Cholesterol** | *0.450* | *0.0126* |
| **IL-10 X IL-4** | *0.374* | *0.0416* | **IL-4 Total Protein** | *-0.506* | *0.0043* | **HDL X IL-4** | *-0.404* | *0.0270* |
| **IL-10 X TNF-α** | *0.410* | *0.0243* | **IL-4 X Albumin** | *-0.371* | *0.0433* | **HDL X Peroxide** | *0.395* | *0.0307* |
| **IL-2 X HDL** | *0.439* | *0.0152* | **IL-4 X IL-2** | *0.435* | *0.0162* | **IL-4 X IL-2** | *0.682* | *<0.0001* |
| **IL-4 X IFN-γ** | *0.642* | *0.0001* | **IL-4 X IL-6** | *0.547* | *0.0018* | **IL-4 X IL-5** | *0.374* | *0.0415* |
| **IL-4 X IL-2** | *0.472* | *0.0084* | **WRS X HDL** | *0.408* | *0.0252* | **IL-4 X TNF-α** | *0.605* | *0.0004* |
| **IL-4 X TNF-α** | *0.599* | *0.0005* | **WRS X Peroxide** | *0.410* | *0.0245* | **IL-5 X TNF-α** | *0.518* | *0.033* |
| **IL-5 X IFN-γ** | *0.586* | *0.0007* | **Peroxide X Triglycerides** | *0.504* | *0.0045* | **LDL X IL-2** | *-0.434* | *0.0165* |
| **IL-5 X TNF- α** | *0.568* | *0.0011* | **4FPTA X HDL** | *-0.389* | *0.0336* | **LDL X IL-4** | *-0.438* | *0.0155* |
| **LDL X IL-6** | *-0.395* | *0.0308* | **4FPTA X WRS** | *-0.939* | *<0.0001* | **LDL X IL-6** | *-0.413* | *0.0232* |
| **4FPTA X WRS** | *-0.947* | *<0.0001* | **4FPTA X Peroxide** | *-0.446* | *0.0135* | **4FPTA X WRS** | *-0.935* | *<0.0001* |
| **TNF-α X BMI** | *0.377* | *0.0437* | **4FPTA X Triglycerides** | *-0.400* | *0.0284* | **TNF-α X IL-2** | *0.363* | *0.048* |
| **TNF-α X IFN-γ** | *0.492* | *0.0058* | **TNF-Α X BMI** | *-0.412* | *0.0265* | **Triglycerides X Peroxide** | *0.362* | *0.0497* |
| **Triglycerides X Age** | *0.397* | *0.0300* | **Total Protein X Fructosamine** | *-0,386* | *0,0353* | **Uric Acid X IL-1F7** | *0.404* | *0.0266* |
| **Triglycerides X IL-5** | *0.442* | *0.0146* | **Total Protein X IL-4** | *-0.506* | *0.0043* |  |  |  |
| **Triglycerides X IL-6** | *0.668* | *<0.0001* | **Total Protein X IL-6** | *-0.361* | *0.0499* |  |  |  |
| **Triglycerides X Peroxide** | *0.451* | *0.0123* | **Total Protein X LDL** | *0.378* | *0.0394* |  |  |  |
| **Uric Acid X Adiponectin** | *-0.497* | *0.0052* | **Triglycerides X IL-6** | *0.446* | *0.0135* |  |  |  |
| **Uric Acid X IL-5** | *-0.337* | *0.0359* | **Uric Acid X IL-5** | *-0.478* | *0.0075* |  |  |  |
|  |  |  | **Uric Acid X TNF-α** | *-0.364* | *0.0483* |  |  |  |

Values of p<0.05 were considered significant.

**Pearson's correlation coefficient of all parameters studied in patients with Sudden Sensorineural Hearing Loss.**
